# Microstrip Patch Sensor for Characterizing Saline Solution Based on Complimentary Split-Ring Resonators (SC-SRRs)

**DOI:** 10.3390/s25072319

**Published:** 2025-04-05

**Authors:** Hussein Jasim, Sadiq Ahmed, Iulia Andreea Mocanu, Amer Abbood Al-Behadili

**Affiliations:** 1 Electrical Engineering, College of Engineering, Mustansiriyah University, Baghdad 00964, Iraq; husseinj95@uomustansiriyah.edu.iq (H.J.); drsadiq18@uomustansiriyah.edu.iq (S.A.); amer_osman@uomustansiriyah.edu.iq (A.A.A.-B.); 2 Department of Telecommunication, Faculty of Electronics, Telecommunications and Information Technology, National University of Science and Technology Politehnica Bucharest, 060042 Bucharest, Romania

**Keywords:** microstrip patch sensor, single complementary split-ring resonance, complex permittivity, parts per thousand (ppt), conductivity

## Abstract

This article presents a novel microstrip patch sensor featuring four rectangular rings represented by single complementary split-ring resonance (SC-SRR) to calculate the complex permittivity of saline solutions within the range of 0 ppt to 100 ppt. This sensor operates via the turbulence technique, utilizing its resonant properties as indicators to find the parameters of the liquid under test (LUT), which arise due to the variations in the salt concentration altering the complex permittivity. This alteration influences the resonant frequency (*f*_r_), reflection coefficient (S_11_), and quality factor (Q). The sensor was designed by using a high-frequency structure simulator (HFSS) and by using an FR-4 substrate and a Teflon box with a height of 1.4 mm and 13.7 mm, respectively. The values of S_11_ at resonance frequency were −34.48 dB, and 2.1328 GHz, respectively. A computer numerical control (CNC) machine was used to fabricate the sensor and Teflon box, and the Teflon box was situated above the four rings to create a strong interaction between the induced electric field and the LUT, thereby achieving high sensitivity in a non-contacting and non-destructive manner. The measurement and simulation results were consistent and aligned with those of Klien and Meissner (in comparison to the theoretical values derived from the single and double Debye models). We derived numerical equations for the conductivity (S/m), dielectric constant permittivity, and concentrations (ppt) using curve fitting origin software, and the results are in good agreement. Due to its performance, we expect that the proposed sensor could be used in agricultural applications to identify freshwater and in medical applications to detect the concentration of salt in saliva or blood and to identify diseases, in addition to many other applications involving mixed liquids.

## 1. Introduction

The salinity of water sources plays a role in assessing environmental quality, since high salinity adversely impacts human and animal health and degrades soil quality. Consequently, the swift assessment of salinity is necessary to address this situation. To overcome these problems, compact and portable salinity sensors have been developed, such as microstrip patch antennas (MPAs). These sensors are based on either the alteration in an electromagnetic field for measuring water conductivity [1] or the alteration in an electromagnetic field for measuring water permittivity [2]. Recently, studies have been conducted on detecting the permittivity of liquids by measuring their microwave frequency performances [3,4,5].

Microstrip patch sensors (MPSs) are an advanced technology that are widely utilized for material characterization due to their efficiency, compact profile, small size, and versatility [6]. In addition, they have a narrow bandwidth, and thus a high quality, which is important for measuring physical quantity when used as a sensor. These sensors are primarily based on microwave engineering principles and are capable of measuring the various physical and chemical properties of materials. The working mechanism involves changes in the electromagnetic properties of the sensor when it interacts with different materials, making it ideal for diverse applications such as industrial quality control, biomedical diagnostics, and environmental monitoring.

MPAs are designed with different shapes, including H, L, M, T, and U shapes, and there are also MPAs with slots, slits, and notches [7,8,9,10,11]. Moreover, the appropriate shape is determined by the application for which the antenna is designed. MPAs have been used in many applications, including detecting the moisture content of leaves [12] and detecting the moisture content in soil [13]. However, an increase in moisture content means an increase in the percentage of water, which thus causes an increase in the relative permittivity and a decrease in the resonant frequency because water molecules absorb electromagnetic radiation. There has also been a study in which an MPS was used for broken rice detection [14]. On the medical side, MPAs have also been used to treat breast cancer using electromagnetic waves [15]; the basic idea of using MPAs is to focus electromagnetic radiation at the tumor site to treat it with hyperthermia. From an agricultural point of view, agriculture depends mainly on two important factors: water and soil. Most countries’ main economy is dependent on agriculture; therefore, salt water and salty soil lead to the collapse of agriculture, and as the reason for the formation of salty soil is the salty water with which the soil is watered, it is necessary to provide an easy, cheap, suitable, and simple method for detecting water salinity [16].

In recent years, microstrip patch antennas have been gaining popularity as a new method to determine the complex permittivity of materials [17]. The MPS method was used to measure the dose of chlorine added to water requiring disinfection [18]. Many configurations of MPAs are used to construct salinity sensors; the defective ground surface technique (DGS) was used to improve the performance of an antenna and reduce its size [19]; the electromagnetic band gap structure (EBG) and coplanar waveguide (CPW) methods were used to detect liquids with different permittivity values by the deposition of the liquid through grooves that existed between the EBG cells [20]. However, because the fluids pass through the center of the antenna, this can lead to corrosion or degradation of the antenna. As reported in [21], the antennae that are used to detect concentrations should be wideband so that they can detect frequency shifts due to changes in the dielectric property of the solution. However, the results show that as the concentration increases, the S_11_ and *f*_r_ values decrease.

Multiple methods exist for measuring the permittivity of a liquid under test (LUT) in order to examine the interaction between the liquid and electric fields and subsequently to extract the characteristics of the dielectric liquid, such as introducing microstrip patch antennas into the LUT, as introduced in [22,23], in which the LUT is placed above the microstrip patch antenna [24], using etched microfluidic channels on substrate materials and passing them through the LUT [25], and using textiles as a substrate material to absorb LUTs with different concentrations [26].

Researchers have devised many methodologies to characterize the complicated permittivity of liquids, and they have been classified as transfer methods and resonance methods. Resonance methods are favored over transfer methods due to their enhanced accuracy, so several resonators for fluid characterization, including the split-ring resonator (SRR) [27], complementary split-ring resonator (CSRR) [28], multiple complementary split-ring resonator (MCSRR) [29], cavity resonator [30], Hilbert structure [31], and Minkowski structure [32], have been proposed and used. The complementary split-ring resonator has gathered significant attention due to its high-quality factor and the ability to concentrate electromagnetic fields effectively.

This work proposes a high-quality factor sensor to measure the dielectric properties of saline solutions, where the salt concentration in freshwater ranges from 0 ppt to 100 ppt. A highly uniform electric field with extremely high density is formed and interacts with the LUT when introduced in the Teflon box, which leads to changes in *f*_r_, S_11_, and Q. Then, the complex permittivity of the LUT can be determined. When compared to previously published sensors, the proposed sensor in our study demonstrates distinct advantages in terms of compactness, repeatability, and accuracy in measuring salinity at high concentrations, using a precise design of a microstrip patch with a single split-ring resonator (SC-SRR).

## 2. Permittivity

Permittivity (ε) refers to the electrical response of a material, meaning the material’s ability to store electrical energy within itself when exposed to an external electric field. Primarily, it measures how easily the material can be polarized [33].

(1) $$\varepsilon =\varepsilon_{o}\varepsilon_{r}$$

where vacuum permittivity ($\varepsilon_{o}$) is equal to 8.85 × 10^−12^ F/m, and relative permittivity ($\varepsilon_{r}$) depends on the material $\varepsilon_{r}$. The relative permittivity is a complex value consisting of a real part (${\varepsilon_{r}}^{\prime }$) and an imaginary part (${\varepsilon_{r}}^{\prime \prime }$).

(2) $$\varepsilon_{r}=\varepsilon_{r}\prime -j\varepsilon_{r}\prime \prime$$

The dielectric constant permittivity, $\varepsilon_{r}\prime$ represents the material’s ability to store energy reversibly. It signifies how much of the applied electric field energy is effectively stored within the material.

Loss factor permittivity, $\varepsilon_{r}\prime \prime$ known as the loss factor, quantifies the energy dissipated or lost as heat within the material due to its interaction with the electric field.

It’s crucial to know that permittivity is not a fixed property. It can change depending on frequency, temperature (T), and salinity (S).

Many authors have studied the complex permittivity of seawater at different frequencies, temperatures, and salinities (20 ppt to 40 ppt); among these, it is worth mentioning Strogren et al. [34,35], Klein and Swift [36], Ellison et al. [37], and Meissner and Wentz [38]. Strogren et al. (1971) [34] conducted seawater measurements utilizing a single Debye model and derived straightforward analytic equations that proved to match well with the measurements. In 1995, a double Debye model was devised to fit the data more accurately at high frequencies when determining Debye parameters. Klein and Swift (1977) were pioneers in formulating a model function for seawater, utilizing data collected by Ho et al. [39,40] to calculate Debye’s parameters. Ellison et al. (1996) [37] utilized their own observations regarding seawater together with Debye’s model to develop a distinct model function. Meissner and Wentz (2004) [38] inverted satellite data to derive a dielectric constant for the sea surface, and from these data, together with contributions from other colleagues, they established a double Debye model function.

In this work, the Klein and Swift model will be used as a reference to compare the performance of the proposed sensor with other works. The Klein and Swift model employs the Debye equation presented in (3) to determine the necessary parameters.

(3) $$\varepsilon =\varepsilon_{\infty }+\frac{\varepsilon_{s}-\varepsilon_{\infty }}{1+j\omega \tau }-j\frac{\sigma }{\omega \varepsilon_{o}}$$

where ω=2πf is radian frequency with f in hertz, ε is complex permittivity, ε_∞_ is dielectric constant at infinite frequency, ε_s_ is static dielectric constant, ε_o_ is permittivity of free space, τ is relaxation time, and σ is ionic conductivity.

## 3. Operational Principle

The general equations for the complex permittivity which give a relation between the variation in the resonant frequency and the permittivity and permeability of the material being tested are given in (4) [41,42].

(4) $$\frac{\Delta f_{r}}{f_{r}}=\frac{\int_{V_{c}}(\Delta \varepsilon E_{1}E_{o}+\Delta \mu H_{1}H_{o})dv}{\int_{V_{c}}(\varepsilon_{o}{\left|E_{o}\right|}^{2}+\mu_{o}{\left|H_{o}\right|}^{2})dv}$$

where ∆f_r_ is the change in resonant frequency, V_c_ is the cavity volume, ∆ε is the change in complex permittivity, ∆μ is the change in complex permeability, ε_o_ is the permittivity of free space, μ_o_ is the permeability of free space, E_o_ is the electric field of the empty cavity, H_o_ is the magnetic field of the empty cavity, E_1_ is the electric field under load condition, and H_1_ is the magnetic field under load condition.

At the resonance frequency, the energy of the electric field is the same as the magnetic field energy stored in the resonator. The resonance frequency alters as the material engages with the stored electric and magnetic field energy or when there is a disturbance in the field distribution. These formulas are valid for guided wave cavities, where the derivation of electromagnetic field expressions is straightforward, according to the effect of the material’s perturbation. Nevertheless, their application in SC-SRR constructions is difficult; so, to precisely assess and manipulate numerous aspects, it is more suitable to create numerical equations for this category of resonators.

It is worth noting that this work focuses on calculating the complex permittivity of freshwater as well as for saline solutions. Therefore, it should be noted that the relative permeability of air is 1, while the relative permeability of water is 0.999, indicating that the water is a diamagnetic material. On the other hand, due to water’s high permittivity, the sensor must be used so that it produces an appropriate electric field which will interact with the permittivity instead of the permeability.

## 4. Antenna Structure

There are many shapes of MPA, and the rectangular one was chosen because it is easy to manufacture, gives good results, and is better at measuring changes in liquid properties than other forms [42]. In this paper, a rectangular microstrip patch antenna operating at a resonance frequency of 2.45 GHz is designed using an FR-4 substrate with a thickness of 1.4 mm, based on the equations in [43].

(5) $$W_{p}=\frac{C}{2f_{r}}\times \sqrt{\frac{2}{\varepsilon_{r}+1}}$$

(6) $$\varepsilon_{\mathrm{eff}}=\frac{\varepsilon_{r}+1}{2}+\frac{\varepsilon_{r}-1}{2}\times {\left(1+12\frac{h}{W_{p}}\right)}^{-0.5}$$

(7) $$\Delta L=0.412h\frac{\varepsilon_{\mathrm{eff}}+0.3}{\varepsilon_{\mathrm{eff}}-0.258}\times \frac{\frac{W_{p}}{h}+0.264}{\frac{W_{p}}{h}+0.8}$$

(8) $$L_{\mathrm{eff}}=\frac{C}{2f_{r}\sqrt{\varepsilon_{\mathrm{eff}}}}$$

(9) $$L_{p}=L_{\mathrm{eff}}-2\Delta L$$

where W_p_ is the width of patch, C is the speed of light 3 × 10^8^ m/s, f_r_ is the resonance frequency, ε_r_ is the relative permittivity of FR-4 (4.4), h is the thickness of the substrate, ε_eff_ is the effective dielectric permittivity, ∆L is the extension length because of the effect of the fringing field, L_eff_ is the effective length (electrical length), and L_p_ is the length of the patch (physical length).

## 5. Sensor Configuration

**Figure 1:**
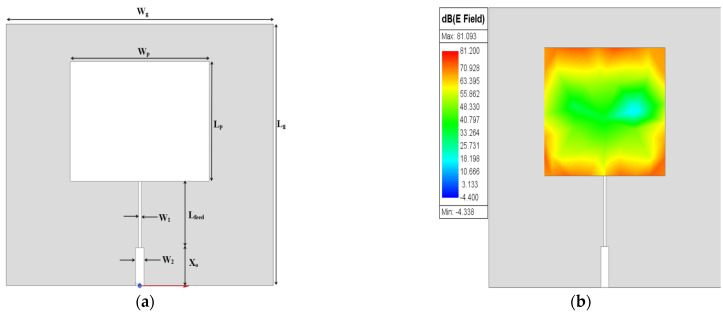
Conventional MPA: (**a**) 2D structure; (**b**) electric field.

The design of the proposed MPS is obtained through three stages. First, the conventional rectangular MPA (L_p_ × W*_p_*) is designed at 2.45 GHz resonance frequency on an FR-4 epoxy dielectric substrate (ε_r_ = 4.4 with h = 1.4 mm and tanδ = 0.02). A microstrip feed line with a quarter-wavelength transformer (λ_g_/4) is used for exciting the patch. The prototype design of an MPA is given in Figure 1a.

The *f_r_* of the patch resonator is designed at (λ/2), and as a result, the maximum distribution of the electric field is concentrated on both edges opposite the feeding port of the patch, as shown in Figure 1b, while the minimum electric field distribution appears in the middle. Optimized dimensions of the rectangular MPA are depicted in Table 1.

**Figure 2:**
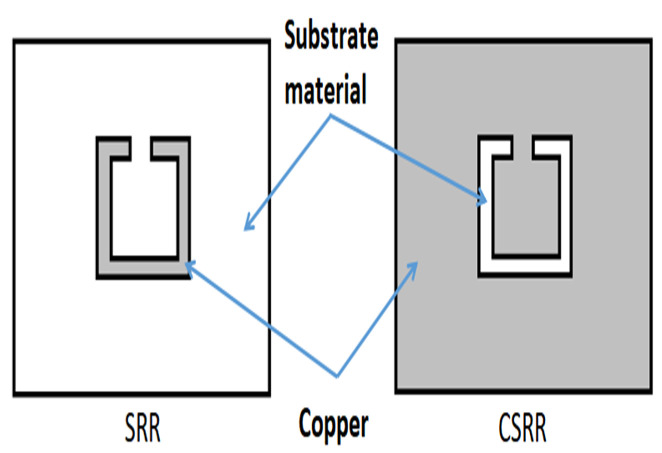
Difference between split-ring resonator (SRR) and complimentary split-ring resonator (CSRR).

In the second stage of the design, a single complementary split-ring resonator (SC-SRR) is etched in the antenna patch. Figure 2 shows the difference between SRR and CSRR. The SC-SRR is designed with a square shape as the complementary split-ring resonator with a length of approximately λ_o_/4 to create a sensitive slot used for sensing the influence of salt concentration in solutions, which reads as a shift in *f*_r_ as well as in S_11_.

**Figure 3:**
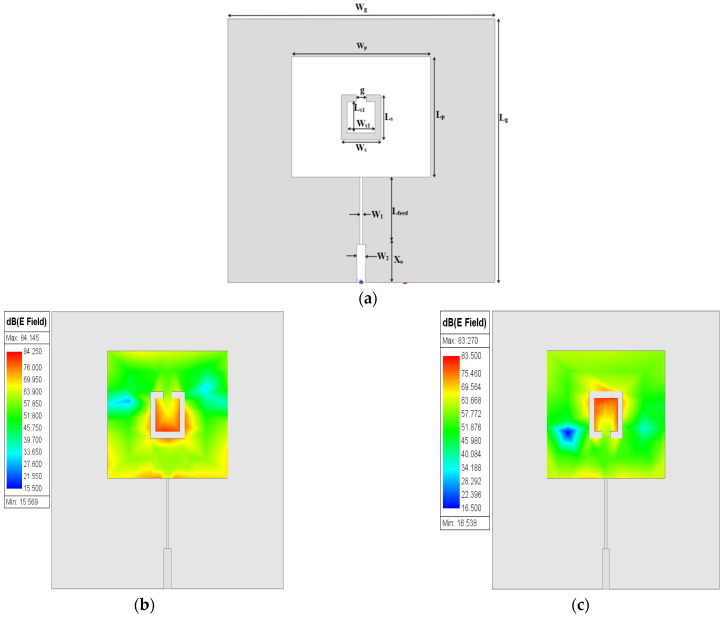

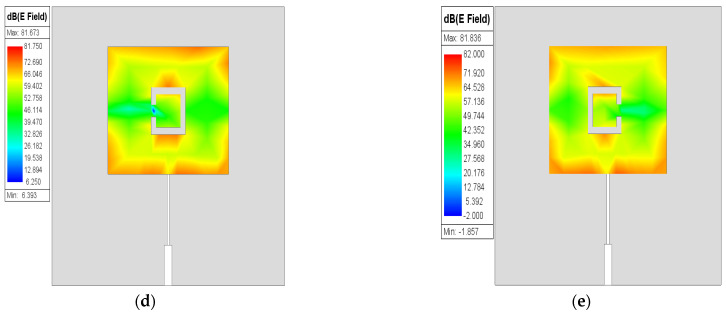
One SC-SRR: (**a**) 2D structure; (**b**) electric field of up SC-SRR; (**c**) electric field of down SC-SRR; (**d**) electric field of left SC-SRR; (**e**) electric field of right SC-SRR.

The second layout stage of the proposed sensor is shown in Figure 3a. The orientation effect of the discrete part of SC-SRR on the electric field distribution on the patch surface is investigated as shown in Figure 3b–e. It can be seen in Figure 3b,c that the maximum surface electric field is concentrated around the slot due to the resulting capacitive effect (open circuit). In Figure 3d,e, it can be observed that most of the distribution of the electric field is concentrated at the edges of the patch surface. This is due to a change in orientation of the discrete part of the SC-SRR. The optimized dimensions of the single SC-SRR added to the conventional MPA are depicted in Table 2.

**Figure 4:**
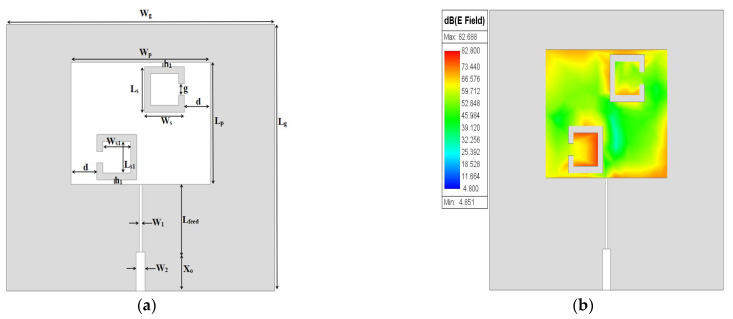
Two SC-SRRs: (**a**) 2D structure; (**b**) electric field.

Due to the above investigation, two SC-SRRs are proposed with opposite orientations, as shown in Figure 4, which depicts most of the electric field distribution at opposite corners of the patch surface, as well as the circumference of the SC-SRRs. However, the intensification of the distribution of the electric field magnitude still needs to be improved further throughout the surface of the patch. The optimized dimensions of the two SC-SRRs added to the conventional MPA are depicted in Table 3.

**Figure 5:**
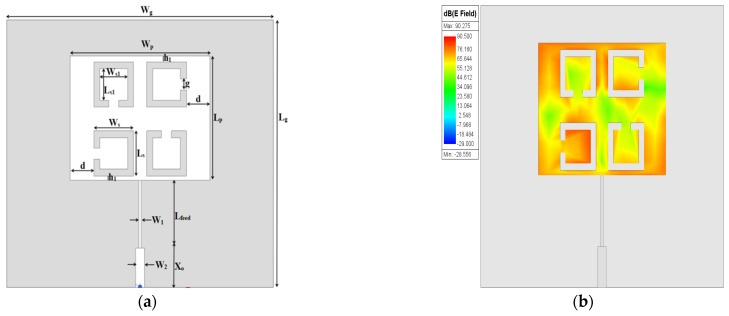
Four SC-SRRs: (**a**) 2D structure; (**b**) electric field.

Finally, four SC-SRRs with different orientations are proposed, as shown in Figure 5, where the distribution of the electric field magnitude is highly intensified at the opposite corner edges of the patch, as well as the circumference of the four SC-SRRs that mediate the center of the patch, therefore allowing the electric field to have stronger intensity among the largest part of the surface parts of the patch resonator. The dimensions of the four SC-SRRs added to the conventional MPA are depicted in Table 4.

**Figure 6:**
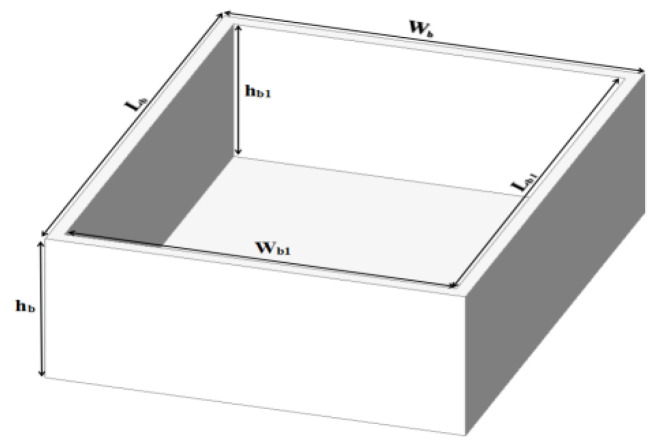
Three-dimensional structure of the container box.

The saline sensitivity performance of the proposed sensor was tested by changing the saline solutions from freshwater to brine (0–100 ppt) by changing the concentration of salt. Saline solutions in contact with the sensor can corrode the copper parts and reduce the sensitivity of the sensor, so to avoid that, the saline solutions must be placed in a container. The Teflon material was chosen to fabricate the container due to its desirable electrical and mechanical properties. The container box (ε_r_ = 2.1 and tanδ = 0.0004) is placed over the patch, covering all four elements of the SC-SRR. The layout and dimensions of the container box are shown in Figure 6 and Table 5, respectively.

**Figure 7:**
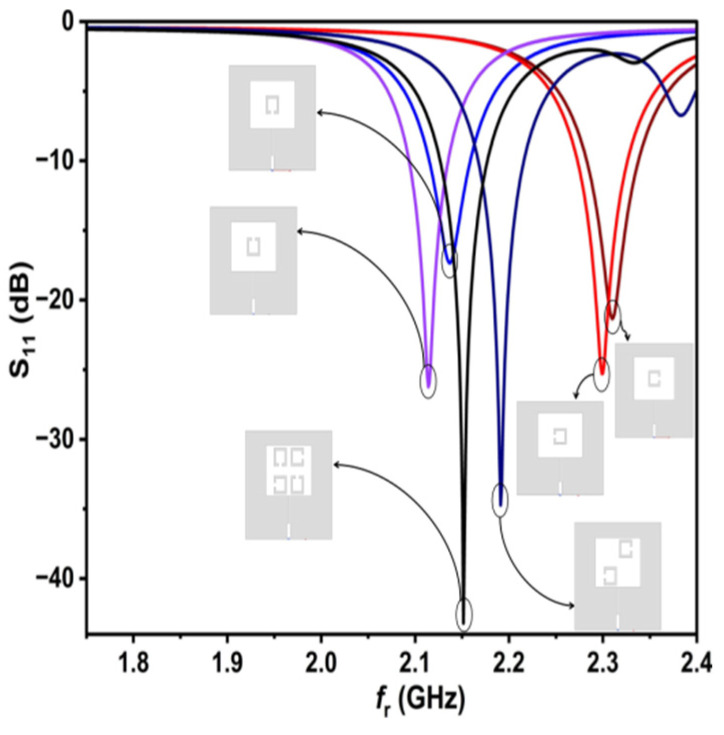
Tested cases by simulation for all designs using HFSS.

Figure 7 illustrates the f_r_ and S_11_ for all designs, starting from the single SC-SRR to the four SC-SRR design. It shows that the four SC-SRR design has the lowest S_11_ and the highest Q. When testing the designs with all samples, the four SC-SRR design was the best among them.

## 6. Sensor Performance

**Figure 8:**
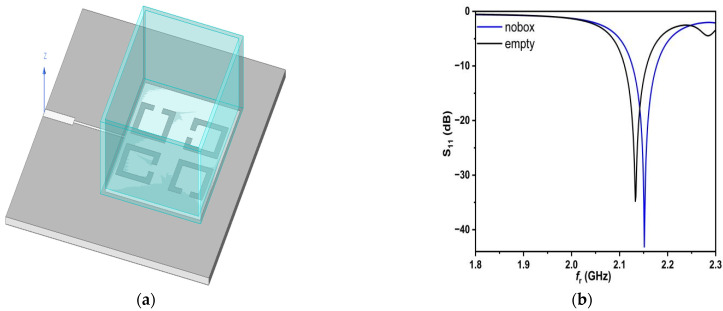
Simulation using HFSS for the proposed sensor: (**a**) four SC-SRRs; (**b**) frequency response.

The four SC-SRRs consisting of symmetrical rings are excited by applying an electric field and with the help of a feed line. Thus, when the container box is placed above the patch which surrounds the four SC-SRRs, the electric field is perturbed, and this effect is reflected in the change of S_11_, f_r_, and Q, as shown in Figure 8.

Analyzing the results in Figure 8, it can be seen that the resonant frequency changes when the box is placed because of Teflon’s permittivity and the variation in the resonant frequency according to relation (5) and depending on Q.

In order to characterize any saline solution tested using the proposed sensor, a numerical model must be derived from the simulation data, which includes f_r_, S_11_, and Q, and thus, a solution for the complex permittivity value can be obtained, as well as the saline concentration. The values for f_r_, Q, and bandwidth (BW) are 2.1515 GHz, 2151.5, and 50 MHz in the case of no box, respectively. The values above in the case of the Teflon box will be 2.1328 GHz, 1066.4, and 46 MHz.

**Figure 9:**
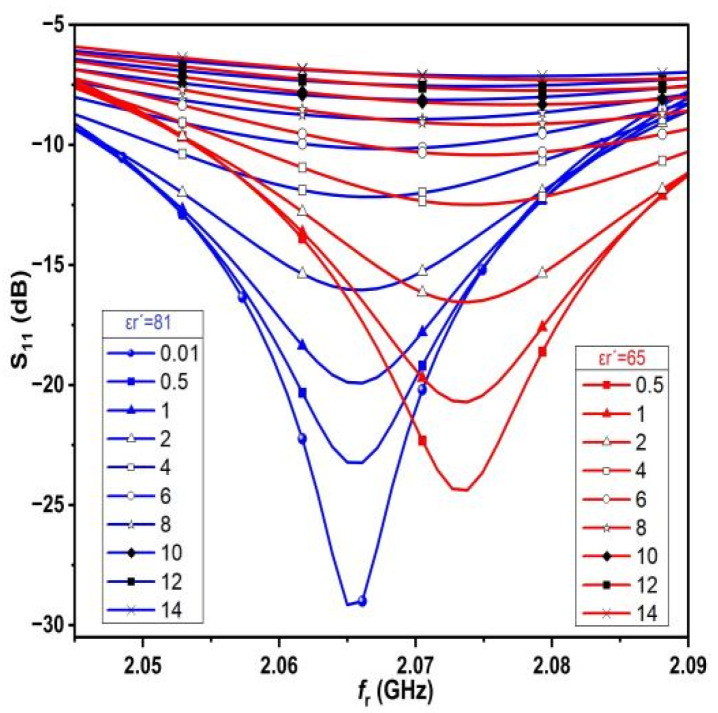
Tested simulation cases using HFSS for each ε_r_′ at different σ.

In order to investigate the performance of the proposed sensor, Figure 9 shows the change in resonance frequency of the proposed sensor, as well as the S_11_ change as a result of testing different values of saline solutions with different dielectric constant permittivity (varying from ε_r_′ = 65 to ε_r_′ = 81). For each value of the dielectric constant permittivity, the conductivity varies from 0.5 S/m to 14 S/m.

## 7. Numerical Modeling

In order to determine the dielectric constant permittivity for each saline solution, several values of ε_r_′ are considered starting from 81 to 65, with each value of ε_r_′ corresponding to different values of conductivity, and with σ ranging from 0.5 S/m to 14 S/m, as shown in Figure 9. The above results are obtained because different salt concentrations in freshwater lead to a change in the dielectric properties, i.e., adding salt to freshwater with different concentrations leads to an increase in the conductivity, σ, of the water. This is clear because it is known that salt water has higher conductivity than freshwater because of the presence of dissolved ions in the solution, which contributes to the process of transferring charges from one place to another.

**Figure 10:**
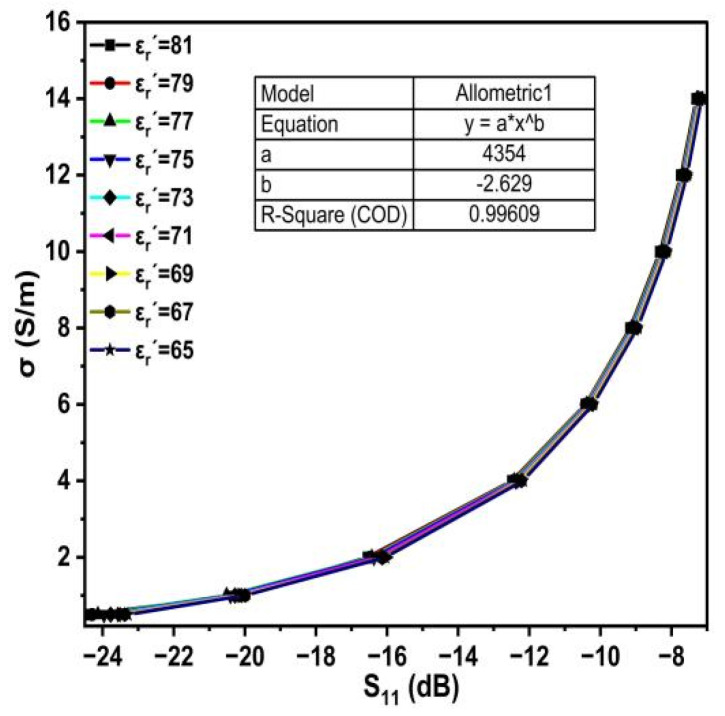
Relationship between S_11_ and the σ for different ε_r_′.

Figure 10 shows the S_11_ extracted from the simulation data (see Figure 9) and plotted with the conductivity for different dielectric constant permittivity. It is clear that the relationship between S_11_ magnitude and conductivity is an exponential relationship, an increase in the slope of the curve can be observed when salt concentrations are increased, and the change in dielectric constant permittivities for different conductivity values is almost constant. The mathematical expression is given in (10).

(10) $$\sigma =4354\times {\left(s_{11}\right)}^{-2.629}$$

After computing the conductivity for all concentrations using the S_11_ values, the second step is to find the dielectric constant permittivity.

As we mentioned earlier, adding salt to water leads to the appearance of dissolved ions in the water, and as a consequence, the free molecules in the water decrease, so these ions cannot store the electrical energy in the way that free molecules do, i.e., the dielectric constant decreases.

**Figure 11:**
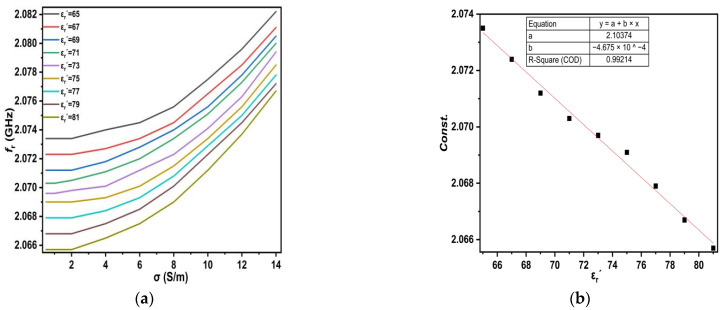
(**a**) The change in f_r_ for different conductivities; (**b**) constant value with dielectric constant permittivity.

In this way, increasing salt concentrations leads to a reduction in the dielectric constant and a shift in the resonant frequency to the right (see Figure 9). The change in dielectric constant permittivity in the range from (65 ≤ ε_r_′ ≤ 81), which corresponds to conductivity range (0.5 ≤ σ ≤ 14), was considered for each dielectric constant permittivity, as shown in Figure 11.

Observing the curves in Figure 11a for each ε_r_′, we notice that they look similar, and the difference is the amount of upward shift. The equations for each curve’s slope for every ε_r_′ are shown in Table 6.

Therefore, the equations shown in Table 6 appear to be similar except for the value of the constant, so they can be rewritten as one standardized equation, as shown in (11).

(11) $$f_{r}=6\times 10^{-5}\sigma^{2}-1\times 10^{-4}\sigma +\mathrm{const}.$$

This constant expression is then taken for each ε_r_′, as shown in Figure 11b, and the equation is as given in (12).

(12) $$\mathrm{const}.=2.1037-4.675\times 10^{-4}{\varepsilon_{r}}^{\prime }$$

Substituting the equations given in (11) and (12), we get the equation given in (13):

(13) $$\varepsilon_{r}\prime =\frac{2.1037+(6\times 10^{-5})\times \sigma^{2}-(10^{-4})\times \sigma -f_{r}}{\left(4.675\times 10^{-4}\right)}$$

The above numerical expression was generated using a curve fitting technique that provides a numerical model for the proposed sensor to calculate the dielectric constant permittivity of the LUTs based on the resonant frequency and the conductivity of each LUT. It was investigated for all values, and the results were in very good agreement.

From [44], the loss factor permittivity can be determined by the following:

(14) $$Q=\frac{{\varepsilon_{r}}^{\prime }}{{\varepsilon_{r}}^{\prime \prime }}$$

The ε_r_″ for all salt concentrations can measured based on Equation (14). The complex permittivity of all LUTs may now be determined.

## 8. Saline Solution Concentrations

There are three types of salt: seawater salt, rock salt, and organic salt. Seawater salts and rock salts represent the source of natural salts that are formed by nature without human intervention, whereas organic salts are salts that have some organic substances added to them, such as iodine.

Salt is an ionic compound that is soluble in water, and the speed at which it dissolves depends on the purity and temperature of the water. The effect of the temperature over the speed of dissolution is direct, so the higher the water temperature, the faster the dissolution process. In this research, we use samples of freshwater with different concentrations of pure sea salt, where relationship (15) is used to find salt concentration.

(15) $$\mathrm{percentage}\;\mathrm{salt}\%=\frac{M_{\mathrm{salt}}}{M_{\mathrm{sea}\_\mathrm{water}}}\times 100$$

where M_salt_ is the mass of salt in grams and M_sea_water_ is the mass of freshwater plus the mass of salt in grams for both. The density of water is given by (16).

(16) $$\rho =\frac{M_{\mathrm{water}}\left(g\right)}{V_{\mathrm{water}}\left(\mathrm{mL}\right)}$$

where ρ is the density of water in g/mL, M_water_ is the mass of water in grams, and V_water_ is the volume of water in ml. The density of water equals 1 g/mL, so substituting it in relation (16), we get relation (17).

(17) $$1g/\mathrm{mL}=\frac{M_{\mathrm{water}}\left(g\right)}{V_{\mathrm{water}}\left(\mathrm{mL}\right)}$$

The mass of water is equal to the volume of water, in the sense of (1 g = 1 mL). So, we can say that the mass and volume of water are directly related in a 1:1 ratio when using grams and milliliters.

The salinity of seawater is measured by parts per thousand (ppt) and denoted by the symbol (‰), which is always used in oceanography as well as in educational exercises.

So, from relationship (15), we find that the salt concentrations can be measured in ppt, as shown in relation 18.

(18) $$\mathrm{ppt}(‰)=\frac{M_{\mathrm{salt}}}{M_{\mathrm{sea}\_\mathrm{water}}}\times 1000$$

Using relation (18), we can calculate the concentrations with high accuracy and without the presence of salt deposits affecting the accuracy of the results.

A freshwater volume of 40 mL was used for all samples where the weight of salt was dissolved in grams to obtain the concentrations (5–100 ppt), as shown in Table 7.

**Figure 12:**
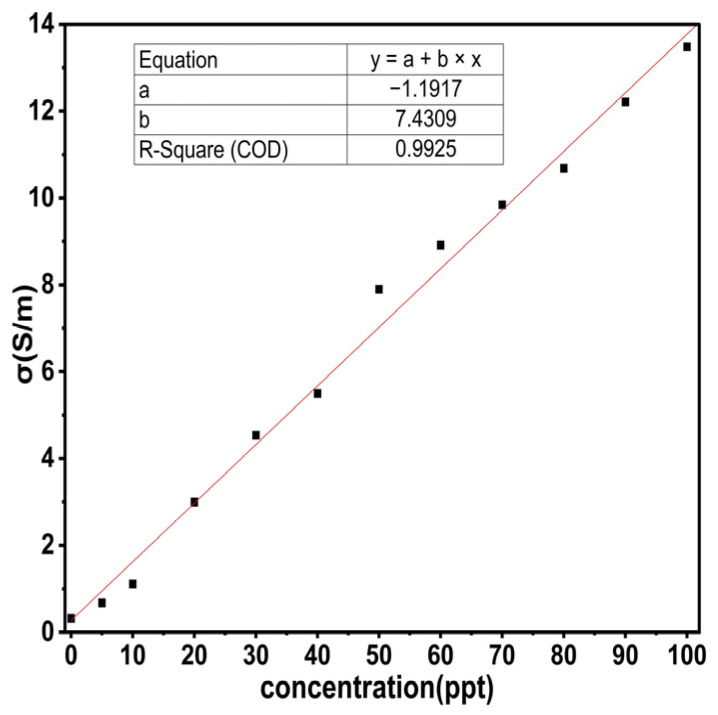
Relation between σ and the concentration.

From Figure 9 and relation (10), both S_11_ and σ can be determined for each saline solution, so it is necessary to derive a numerical relation to calculate the concentrations for each saline solution. Figure 12 shows the curve fitting to find the concentrations for each LUT.

(19) Conc.(οοο)=−1.1917+7.4309σ

Relation (19) was tested for all values, and the results were very close to the theoretical results shown in Table 7.

## 9. Sensor Fabrication and Experimental Setup

The modeling results are verified by fabricating and experimentally testing a prototype sensor. The simulated and measured S_11_ and resonant frequencies are contrasted. The proposed sensor was fabricated using a computer numerical control (CNC) machine by etching copper, which is printed on an FR-4 substrate material.

**Figure 13:**
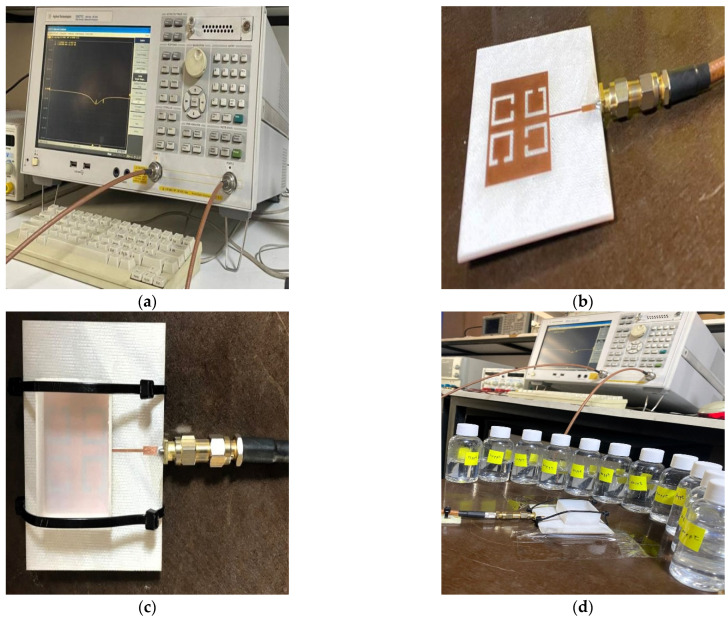
Proposed sensor setup: (**a**) the E5071C Network Analyzer; (**b**) the proposed sensor; (**c**) the proposed sensor and Teflon box above the patch; (**d**) samples (0–100 ppt).

In addition, a Teflon box was also fabricated to act as a container for the LUTs with the dimensions (37 × 29 × 13.7 mm^3^). The proposed sensor, which has dimensions of (67.4 × 59 × 1.4 mm^3^), measures the reflection coefficient and resonance frequency for different concentrations of salt. The proposed sensor was connected to Sub-Miniature version A (SMA) of the Agilent Technologies E5071C Network Analyzer (Agilent Technology, Santa Rosa, USA) from 300 KHz to 20 GHz with 50 Ω of transmission line. The f_r_ and S_11_ are reported by the E5071C Network Analyzer screen for different concentrations of salt (0–100 ppt) in freshwater. As seen in Figure 13c, the proposed sensor has a Teflon box above the patch and is fixed with nylon self-locking to stabilize the readings as well as the weight. The experimental setup is shown in Figure 13.

## 10. Measurement and Result

**Figure 14:**
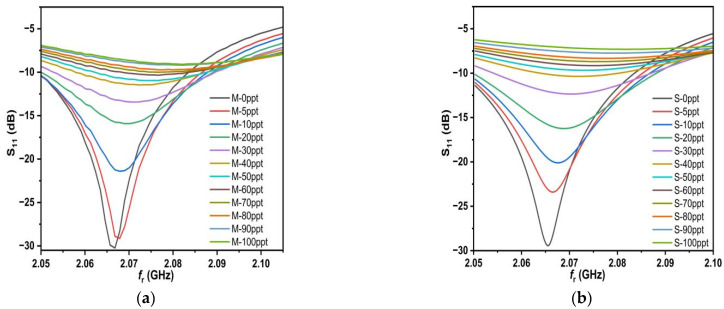
The results: (**a**) measurement; (**b**) simulation.

The female SMA at 50 Ω is connected to the feed line and ground of the proposed sensor through mechanical soldering to measure S_11_ with the help of a network analyzer. The male SMA at 50 Ω is connected to the proposed sensor. The frequency range of the network analyzer was calibrated from 1.75 GHz to 2.3 GHz. After the calibration was set, the samples (0–100 ppt) were placed in a Teflon box with a height of 2 mm, and the data for S_11_ and the resonant frequency of each sample were recorded, as shown in Figure 14a.

**Figure 15:**
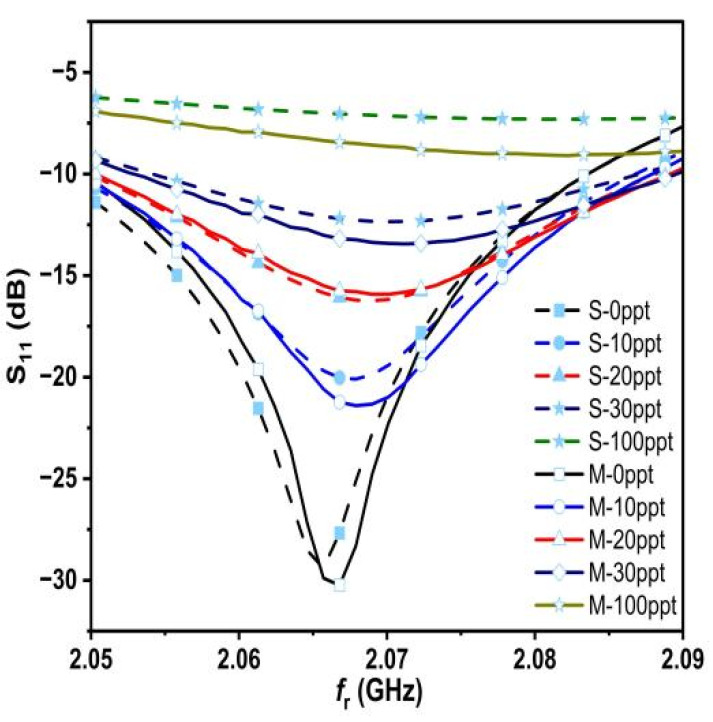
The measured and simulated results.

By observing the measured results, it is clear that as the concentration increases, the value of S_11_ increases as well and the f_r_ shifts to the right and on a regular basis. This means that the proposed sensor has high sensitivity for different salt concentrations. Observing Figure 14, the measured and simulated results from the two figures appear to be quite similar, and this is due to the accuracy of our work in both simulated and measured aspects, especially the accurate and correct sampling. Therefore, we believe that it is necessary to take samples and measure them accurately and correctly and to use the deduced relation 18. In some of the previous literature, we saw a lot of salt deposits in the measured samples, and this leads to inaccurate and degraded measurements. To make the picture clearer, let us combine the measured and simulated results into a single graph, as shown in Figure 15.

The measured quality factor, resonant frequencies, and S_11_ response are compared to simulated values. The discrepancies between the measured and simulated results appear due to various causes, including production tolerances, environmental circumstances, and the network analyzer’s accuracy. Nevertheless, there is a good agreement between the measured and simulated results.

**Figure 16:**
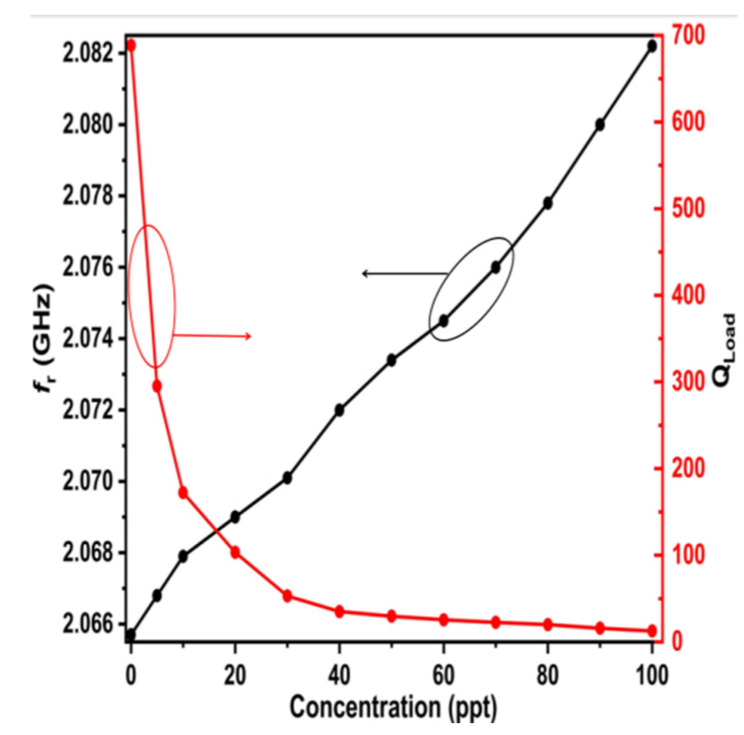
The simulated resonance frequency (black line) and loaded quality factor (red line) of the proposed resonator.

Moreover, the simulated results of the resonant frequency and quality factor for LUTs with different salt concentrations are plotted in Figure 16.

Figure 16 illustrates that the quality factor diminishes to around 700 in the presence of freshwater (down from an initial value of 2151.5) and subsequently declines progressively with rising salt concentrations (red curve). The value of the resonant frequency progressively and precisely rises with increasing concentrations (black curve).

## 11. Results Comparison

The MATLAB software (R2020a) was used to apply the equations for Klein [36] and Meissner [38] to find the conductivity, dielectric constant permittivity, and loss factor permittivity at a frequency of 2.1328 GHz and a room temperature of 25 °C with concentrations of 5, 10, 20, 30, and 40 ppt. The measured results obtained from the proposed sensor were recorded and are shown in Table 8.

To prove the accuracy of the sensor, the measured results of the proposed sensor were compared with the theoretical results of Klein and Meissner, as shown in Table 9. There is a slight difference between our results and those of Klein and Meissner. The discrepancy arises because the materials analyzed by Klein and Meissner were seawater samples, whereas the samples utilized in this research are saline solutions. Ellison et al. [37] observed that the conductivity and permittivity of NaCl solutions and actual seawater exhibit considerable discrepancies at identical temperature and salinity, particularly at frequencies below 3 GHz.

Nortemann stated in [44] that sodium chloride solutions are given as a function of frequency. The imaginary part ε′′ has two contributions, one due to dielectric losses ε_d_′′ and the other due to ion drift losses ε_σ_′′.

(20) $$\varepsilon \prime \prime (f)=\varepsilon_{d}\prime \prime (f)+\varepsilon_{\sigma }\prime \prime (f)$$

(21) $$\varepsilon_{\sigma }\prime \prime =\frac{\sigma }{\omega \varepsilon_{o}}$$

where ω=2πf is the radian frequency with f in hertz, ε′′ is the loss factor permittivity, ε_d_′′ is the dielectric losses, ε_σ_′′ is the ionic drift losses, and ε_o_ is the permittivity of free space.

Ho et al. [39,40] demonstrated that sodium chloride solutions and seawater with identical amounts of salt have different permittivities. Ellison and al. proposed in [37] that to achieve convergence of the results, artificial seawater samples should be created and used for chemical and biological investigations.

## 12. Comparison with Literature


**Ref.**

**Sensor Type**

**Image**

**Substrate**

***f_r_***

**GHz**

**Liquid**

**Type**

**Drawback**
[2]Rectangular with in-set-fed and chamber as container

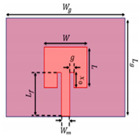

Silicon<3Saline solu-tion- The height of the proposed sensor is high. - The structure of the sensor is difficult to handle when placing or withdrawing samples.[17]Rectangular with in-set-fed thin rectangular and two circular slots

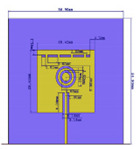

FR-42.33Material and liquid- No fabrication for sensor. - Detected the relative permittivity of the layer as from 1 to 10.[26]Circular ring monopole

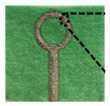

Textile2.4Salt and sugar- This methodology is difficult because it needs conductive yarn and an embroidery machine. - It also needs to be rinsed and left to dry every time it is measured.[19]Defected ground surface technique (DGS)

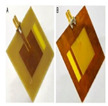

FR-42.4Salt and sugar- The proposed sensor is dipped in solution that may lead to deterioration.
- Too many deposits in the samples.[25]Microfluidic channels are etched on PDMS substrate

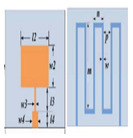

(PDMS)10Liquid- No fabrication for the sensor. - The proposed method can recognize liquids with a permittivity of less than 30.[45]Disposable antenna

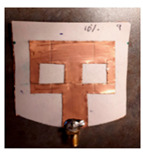

Cellulose paper2 and 3.5Saline solu-tion- The sensor was manufactured by hand using a cutter and scissors, which may result in manufacturing differences. - The number of sensors deployed is equal to the number of samples. No changes occur in the given sensor when absorbing more than 0.4–0.5 mL. - S11 at 2 GHz is less than −10 dB, which may degrade during testing and is difficult to see in the VNA.[5]Inset-fed microstrip patch antenna

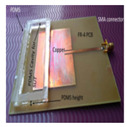

FR-41.45Glucose- Samples are in contact with the sensor, which may lead to the deterioration. - Sample sizes are large, 7.5 mL. - Change in results as the experiment is repeated.

In article [2], silicone was used as a container for saline solutions from 20 ppt to 40 ppt, but silicone naturally absorbs salts, which affects the accuracy of the results, and the structure of the sensor makes it difficult to insert and remove LUT.

In article [17], the sensor was not manufactured, but the researcher claimed that the sensor can measure solids and liquids which have a permittivity of 1 to 10 when placed on the sensor, i.e., in a contact manner.

In [26], the sensor is fabricated using conductive yarns that are sewn onto a textile. This process requires an embroidery machine and secondary materials that are difficult to obtain. In addition, the sensor is rinsed and left to dry for each sample test, which is time-consuming. The concentrations tested for sugar and salt were 5%, 10%, and 20%.

In article [19], the defected ground surface technique is used to improve the performance of the antenna and reduce its size, but we note that its size is not small enough compared to previous research. Furthermore, the immersion of the antenna in the LUT leads to the corrosion of the sensor’s crucial components. The samples tested consist of sugar and salt solutions with concentrations of 20%, 50%, and 80%, but these concentrations suffer from many sediments in the solution, and these sediments cause inaccurate results.

In [25], a sensor is designed with microfluidic channels in the substrate to allow the liquid to pass through them. This method causes bubbles to form inside these channels, affecting the accuracy of the results. The LUTs that were simulated are benzene, acetic acid, alcohol, and water, but the sensitivity decreases with increased permittivity, so it is suitable for liquids with a permittivity of less than 30.

In article [46], a disposable sensor with cellulose paper as a substrate was used due to its liquid-absorbing nature. Scissors and a cutter were used in the production of the sensor, and this method is unsuitable because it produces different resonant frequencies and reflection coefficients. The samples tested were saline solutions with concentrations of 0.05%, 0.5%, and 1%, which represent artificial sweat. Moreover, no changes occur in the given sensor when absorbing more than 0.4–0.5 mL.

In article [5], an inset-fed microstrip patch antenna was used to detect sugar in water. A rectangular cavity made of PDMS is placed in the area where the electric field is concentrated. In this way, the samples come into direct contact with the sensor and may cause the important parts to corrode or damage them. Samples of sugar solutions were used from 0 g/mL to 0.6 g/mL.

In this article, all these flaws were overcome in a non-contact and non-destructive manner, using Teflon, which is characterized by its good mechanical and electrical properties. Moreover, Teflon is naturally non-absorbent to salts, which allows for a very wide range in the repetition of the measurement process, so more than 10 samples of saline solutions from 0 ppt to 100 ppt were tested. All the results were accurate and matched the simulated ones.

## 13. Conclusions

In this paper, a sensor with four single complementary split-ring resonators for microwave characterization of saline solutions (0–100 ppt) has been suggested and investigated. The proposed sensor operates in the frequency range between 1.75 GH and –2.3 GHz. A numerical model was developed to determine the conductivity (σ), dielectric constant permittivity (ε_r_′), and concentrations (ppt) for all saline solutions, as well as a relation for calculating the concentrations of saline solutions with parts per thousand (ppt). The test was performed on several standard samples, and the measured results were very close to the theoretical results of Klein and Meissner. For conductivity, loss factor permittivity, and dielectric constant permittivity, the relative error was less than ±0.3. This sensor is useful because it can be used to determine the complex permittivity of different salt solutions without touching them, damaging them, or costing a lot of money. We believe it can accurately measure the complex permittivity of a wide range of liquids, including methanol, ethanol, chlorine, blood, and saliva.

## Figures and Tables

**Table 1:** The optimized dimensions of the conventional MPA.

Parameters	Value (mm)
h	1.4
ε_r_	4.4
W_p_	35
L_p_	27
L_g_	59
W_g_	67.4
X_o_	8.58
L_feed_	15
W_1_	0.7
W_2_	2.22

**Table 2:** The dimensions of MPA with one SC-SRR.

Parameters	Value (mm)	Parameters	Value (mm)
h	1.4	W_1_	0.7
ε_r_	4.4	W_2_	2.22
Wp	35	Ls	10
Lp	27	Ls_1_	7
Lg	59	Ws	10
Wg	67.4	Ws_1_	7
X_o_	8.58	g	2.5
L_feed_	15		

**Table 3:** The dimensions of MPA with two SC-SRRs.

Parameters	Value (mm)	Parameters	Value (mm)
h	1.4	W_2_	2.22
ε_r_	4.4	L_s_	10
W_p_	35	L_s1_	7
L_p_	27	W_s_	10
L_g_	59	W_s1_	7
W_g_	67.4	g	2.5
X_o_	8.58	h_1_	1
L_feed_	15	d	6.5
W_1_	0.7		

**Table 4:** The dimensions of MPA with four SC-SRRs.

Parameters	Value (mm)	Parameters	Value (mm)
h	1.4	W_2_	2.22
ε_r_	4.4	L_s_	10
W_p_	35	L_s1_	7
L_p_	27	W_s_	10
L_g_	59	W_s1_	7
W_g_	67.4	g	2.5
X_o_	8.58	h_1_	1
L_feed_	15	d	6.5
W_1_	0.7		

**Table 5:** The dimensions of the container.

Parameters	Value (mm)	Parameters	Value (mm)
ε_r_	2.1	W_b_	29
tanδ	0.0004	W_b1_	27
L_b_	37	h_b_	13.7
L_b1_	35	h_b1_	13

**Table 6:** The equations for each ε_r_′.

ε_r_′	Equation	R^2^
65	*f*_r_ = 6 × 10^−5^σ^2^ – 2 × 10^−5^σ + 2.0735	0.9998
67	*f*_r_ = 6 × 10^−5^σ^2^ – 2 × 10^−5^σ + 2.0724	0.9992
69	*f*_r_ = 5.5 × 10^−5^σ^2^ – 7 × 10^−5^σ + 2.0712	0.9995
71	*f*_r_ = 5.5 × 10^−5^σ^2^ – 2 × 10^−5^σ + 2.0703	0.9998
73	*f*_r_ = 6 × 10^−5^σ^2^ – 1 × 10^−4^σ + 2.0657	0.9987
75	*f*_r_ = 6 × 10^−5^σ^2^ – 2 × 10^−5^σ + 2.0691	0.9998
77	*f*_r_ = 6 × 10^−5^σ^2^ – 1 × 10^−4^σ + 2.0679	0.9994
79	*f*_r_ = 5.5 × 10^−5^σ^2^ – 1 × 10^−4^σ + 2.0667	0.9992
81	*f*_r_ = 6 × 10^−5^σ^2^ – 6 × 10^−5^σ + 2.0657	0.9998

R^2^ symbolizes the amount of regression.

**Table 7:** Concentration of saline solution (ppt).

Concentration (‰)	Mass of Salt (g)
5	0.2
10	0.4
20	0.8
30	1.23
40	1.66
50	2
60	2.55
70	3
80	3.47
90	3.95
100	4.43

**Table 8:** The measured results of the proposed sensor.

Concentration (ppt)	*f*_r_ (GHz)	S_11_ (dB)
5	2.0679	−29.133
10	2.0679	−21.403
20	2.069	−15.921
30	2.0712	−13.44
40	2.0723	−12.276
50	2.0745	−10.96
60	2.076	−10.342
70	2.077	−10.006
80	2.078	−9.715
90	2.0811	−9.184
100	2.0822	−9.085

**Table 9:** Comparison between the measured results of the proposed sensor and the theoretical results for both Klein and Meissner.

Conc. (ppt)	σ Klien	σ Meissner	σ Proposed	ε_r_′ Klien	ε_r_′ Meissner	ε_r_′ Proposed	ε_r_′′ Klien	ε_r_′′ Meissner	ε_r_′′ Proposed	Relative Error for σ	Relative Error for ε_r_′	Relative Error for ε_r_′′
5	0.8786	0.8958	0.6152	76.9661	77.138	76.4945	7.4082	7.5531	5.6179	0.3132	0.0083	0.2562
10	1.6987	1.7022	1.3837	75.8539	75.9121	76.5273	14.323	14.353	12.4547	0.1870	0.0081	0.1322
20	3.2126	3.2087	3.0123	73.8322	73.5708	74.7448	27.0884	27.0558	26.8947	0.0611	0.0159	0.0059
30	4.6212	4.6248	4.7024	71.9154	71.3695	71.3509	38.9659	38.9962	41.8498	0.01679	0.0002	0.0731
40	5.9732	5.9727	5.9670	69.9049	69.2998	70.4590	50.3659	50.3612	53.4571	0.0009	0.0167	0.0614
50	-	-	8.0394	-	-	69.0353	-	-	71.4939	-	-	-
60	-	-	9.3646	-	-	68.5034	-	-	83.1606	-	-	-
70	-	-	10.2141	-	-	68.3173	-	-	90.6744	-	-	-
80	-	-	11.0382	-	-	68.2498	-	-	98.1220	-	-	-
90	-	-	12.7960	-	-	66.6198	-	-	113.2824	-	-	-
100	-	-	13.1659	-	-	65.4201	-	-	116.4413	-	-	-

## Data Availability

The data are contained within the article.

## Abbreviations

The following abbreviations are used in this manuscript:

SC-SRR	Single complementary split-ring resonance
CSRR	Complementary split-ring resonator
MCSRR	Multiple complementary split-ring resonator
SRR	Split-ring resonance
ppt	Parts per thousand
Conc.	Concentration
MPA	Microstrip patch antenna
MPS	Microstrip patch sensor
LUT	Liquid under test
MUT	Material under test
